# Active Vision in Driving: Joint Modeling of Scanpaths and Risk Perception

**DOI:** 10.3390/jemr19030059

**Published:** 2026-06-01

**Authors:** Chao Gou, Yueyao Lin, Yuchen Zhou, Wenjie Shi, Jincheng Jiang

**Affiliations:** School of Intelligent Systems Engineering, Sun Yat-sen University, Shenzhen 518107, China; gouchao@mail.sysu.edu.cn (C.G.); linyy99@mail2.sysu.edu.cn (Y.L.); zhouych37@mail2.sysu.edu.cn (Y.Z.); shiwj9@mail2.sysu.edu.cn (W.S.)

**Keywords:** driver scanpath prediction, active vision, visual search, traffic risk perception, adversarial inverse reinforcement learning

## Abstract

Under the Active Vision hypothesis, eye movements are not passive responses to visual stimuli but are actively guided by task demands and internal goals. In driving, scanpaths may therefore reflect an ongoing process of information sampling for risk assessment. However, current computational models often isolate scanpath prediction from risk assessment, overlooking their intrinsic cognitive coupling. In this study, we investigate whether driver scanpaths and traffic risk perception can be jointly modeled within a unified framework. We propose a computational approach based on the introduced **A**dversarial **I**nverse **R**einforcement **L**earning (**AIRL**), where gaze behavior is interpreted as a policy that maximizes a latent safety-related reward. By employing a generator to simulate human-like sequences of fixations and saccades, and a discriminator to approximate the internal reward signal, our framework ensures that generated scanpaths synergistically inform downstream risk perception. To facilitate this research, we constructed the BDDA dataset, aggregating over 13,000 spatio-temporal gaze points with explicit risk annotations to study this joint mechanism. Experimental results indicate that simultaneously modeling the “where” (scanpath dynamics) and the “why” (risk perception) significantly outperforms the compared baseline methods on the proposed BDDA dataset. These findings provide computational evidence for a functional coupling between visual attention and risk perception, supporting the view that eye movements serve as an active mechanism for acquiring task-relevant information in safety-critical environments.

## 1. Introduction

A driver’s scanpath, the sequence of fixations and saccades deployed while navigating traffic, offers a direct window into the cognitive process underlying complex decision-making. It reveals a driver’s focus, information prioritization, and preparedness for evolving situations. Consequently, accurately modeling and predicting these scanpaths is a critical pursuit, holding immense promise for quantifying scene-induced visual load [1], developing proactive safety interventions, and designing more intuitive autonomous vehicles that align with human expectations [2].

Early investigations into driver attention primarily focused on saliency prediction [3,4], which failed to capture the intricate spatio-temporal dependencies of human gaze. More recent data-driven approaches using architectures like LSTMs and Transformers have shown greater promise in learning sequential patterns from large-scale gaze data [5,6,7,8]. However, they still face persistent challenges, including limitations in long-term prediction accuracy, poor generalization across diverse driving contexts, and ineffective integration of multi-modal information such as vehicle dynamics and semantic scene understanding. Crucially, these limitations stem from a fundamental theoretical flaw: existing models often treat gaze as a passive reaction to visual stimuli (bottom-up saliency), failing to account for the active, goal-directed nature of human vision, where attentional resources are allocated based on internal cognitive states rather than just pixel-level features.

**Figure 1:**
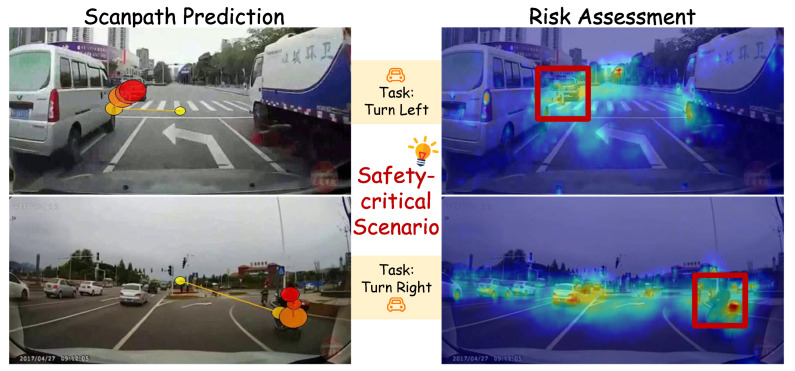
The driver’s scanpath is represented by circles progressing from yellow (initial fixation) to red (final fixation), where larger radii indicate longer fixation durations. It reflects the cognitive process underlying traffic scene perception. In safety-critical scenarios, spatio-temporal modeling of such eye movements contributes to rapid risk assessment.

This theoretical oversight manifests most acutely in the prevailing single-task paradigm. From a cognitive perspective, treating scanpath prediction and risk assessment as isolated tasks creates an artificial dichotomy. According to Active Vision theory, perception is not merely about observing the scene but is actively sampled to serve a downstream task. Consequently, a driver’s attentional allocation is not random; it is intrinsically linked to their continuous assessment of potential hazards and risks. As illustrated in Figure 1, this cognitive process, reflected in their scanpaths, provides substantial evidence for traffic risk assessment. While deep learning approaches for risk assessment are rapidly expanding [9,10,11,12], the potential of jointly modeling scanpath prediction and risk perception simultaneously remains largely unexploited.

In this work, we investigate whether scanpath generation and risk perception can be jointly modeled within a unified computational framework. We propose an approach based on **A**dversarial **I**nverse **R**einforcement **L**earning (**AIRL**), in which gaze behavior is modeled as a policy that maximizes a latent safety-related reward. Within this framework, scanpaths are not only predicted as behavioral outputs but also serve as structured inputs for risk estimation. Our proposed framework focuses on learning not only where a driver will look, but also why, by jointly modeling their visual search strategy and their implicit evaluation of risk. To the best of our knowledge, this is the first attempt to unify scanpath prediction and risk perception within an adversarial reinforcement learning paradigm, explicitly capturing the cognitive dependency between where we look and how we perceive danger. The main contributions of this work are threefold:

- **A Novel AIRL-based Framework:** We propose a generative model that integrates inverse reinforcement learning with adversarial strategies to simulate the “active” nature of driver attention, incorporating top-down task objectives alongside bottom-up visual cues.
- **Cognitive Risk Assessment:** We introduce a multi-task Transformer that models risk not as a static classification, but as a dynamic outcome of the active visual search process, mirroring how humans accumulate evidence to determine safety.
- **Ecological Validation with the BDDA Dataset:** We present the BDDA dataset, a benchmark designed to test the ecological validity of attention models in safety-critical scenarios. Our framework demonstrates superior performance on this benchmark, confirming that explicit modeling of the cognitive link between attention and risk yields more accurate and human-like predictions.

## 2. Related Works

### 2.1. Review of Scanpath Modeling

Early eye movement modeling primarily predicted saliency maps to represent attentional distribution. However, these static heatmaps often overlook the temporal dependencies of vision, i.e., the sequential order in which regions of interest are fixated. Modeling scanpaths is therefore essential for understanding the dynamic nature of human vision. Scanpath modeling generally follows two paradigms: bottom-up and top-down. Bottom-up approaches emphasize the intrinsic attributes of visual stimuli, such as color and shape, which automatically attract attention. Yet, these methods often fail to account for the impact of prior knowledge, context, and specific task requirements on attentional allocation. In contrast, top-down approaches posit that observers allocate attention based on specific goals, such as visual search or visual question answering (VQA).

While early studies utilized mathematical modeling and traditional machine learning to predict free-viewing scanpaths [13,14], the emergence of Convolutional Neural Network (CNN) and Recurrent Neural Network (RNN)-based architectures enabled more effective extraction of spatial features [15,16] and the capturing of long-range temporal dependencies [17,18,19,20]. With the evolution of deep learning, Transformer-based architectures have been introduced to model complex spatial layouts and dynamic mappings. Mondal et al. [21] achieve efficient and stable visual search on the COCO-Search18 dataset using Gazeformer, while Xue et al. [22] proposed a few-shot personalized model that enables adaptation to unseen subjects with minimal support data.

Furthermore, to simulate the interaction between an observer and their environment, Reinforcement Learning (RL) and Inverse Reinforcement Learning (IRL) have been applied to recover the latent reward functions governing gaze policy. Yang et al. [23] modeled viewers’ internal belief states as dynamic contextual belief maps of object locations, which were used to predict human-like target search scanpaths. Huang et al. [24] were the first to apply IRL to traffic scenarios, predicting human scanpaths across different driving tasks through adversarial learning. More recently, diffusion models have been employed to capture the high stochasticity and individual variability inherent in human eye movements [25,26,27]. Despite these advancements, most existing methods treat scanpath prediction as an isolated perceptual task. They overlook the intrinsic cognitive coupling between oculomotor behavior and high-level goals, such as risk perception and safety decision-making in traffic scenarios. Our work addresses this gap by unifying scanpath planning and risk assessment within a single computational framework.

### 2.2. Review of Traffic Risk Assessment

Traffic risk assessment is a fundamental component of intelligent driving systems, aiming to quantify potential hazards and provide a computational basis for safety-critical decision-making. Traditional traffic risk assessment methods typically rely on the physical attributes of roads, vehicles, and the environment, such as vehicle speed, space headway, road conditions, and traffic signals [28,29,30]. Early kinematic indicators like Time-to-Collision (TTC) were widely adopted to identify immediate collision threats based on direct physical distance measurements. However, these traditional methods often struggle with the high complexity, non-linearity, and high dynamics of real-world traffic scenes.

State-of-the-art models have evolved to represent traffic risk as a dynamic outcome of participant interactions. Rather than treating objects in isolation, these frameworks utilize RNNs and Graph Convolutional Networks (GCNs) to model the evolving relational features within the traffic scene. Agarwal et al. [31] introduced a lightweight framework that integrates GNN and long short-term memory (LSTM) layers to embed spatiotemporal scene graphs for efficient and early collision prediction on edge hardware. While previous models focused on visible agent interactions, Wang et al. [32] introduced GSC, a framework that explicitly accounts for occluded agents through graph convolution operations and a novel dynamic adjacency matrix derived from historical trajectories. Song et al. [33] combined global context-enhanced GNNs with temporal attention to identify critical interactions leading to accidents. To bridge the gap between deterministic modeling and behavioral uncertainty, Liu et al. [34] introduced a field-theoretic model that captures multi-agent interactions via interaction fields and probabilistic motion distributions, enabling adaptive risk prediction in long-tail scenarios. Furthermore, some methods like CPTR-LLM [35] employed Large Language Models and a chain-of-thought framework to achieve high-accuracy collision prediction and comprehensive takeover requirement analysis, offering a more cognitive and interpretable approach to road safety. Nevertheless, the aforementioned methods tend to rely on passive feature extraction from the entire visual scene, often overlooking the explicit modeling of the driver’s cognitive process, specifically the active and goal-directed acquisition of safety-critical information. Our framework addresses this critical gap by internalizing the human visual foraging mechanism, where scanpath serves as a high-fidelity information filter to guide the risk perception.

## 3. Method

### 3.1. Cognitive Architecture Overview

**Figure 2:**
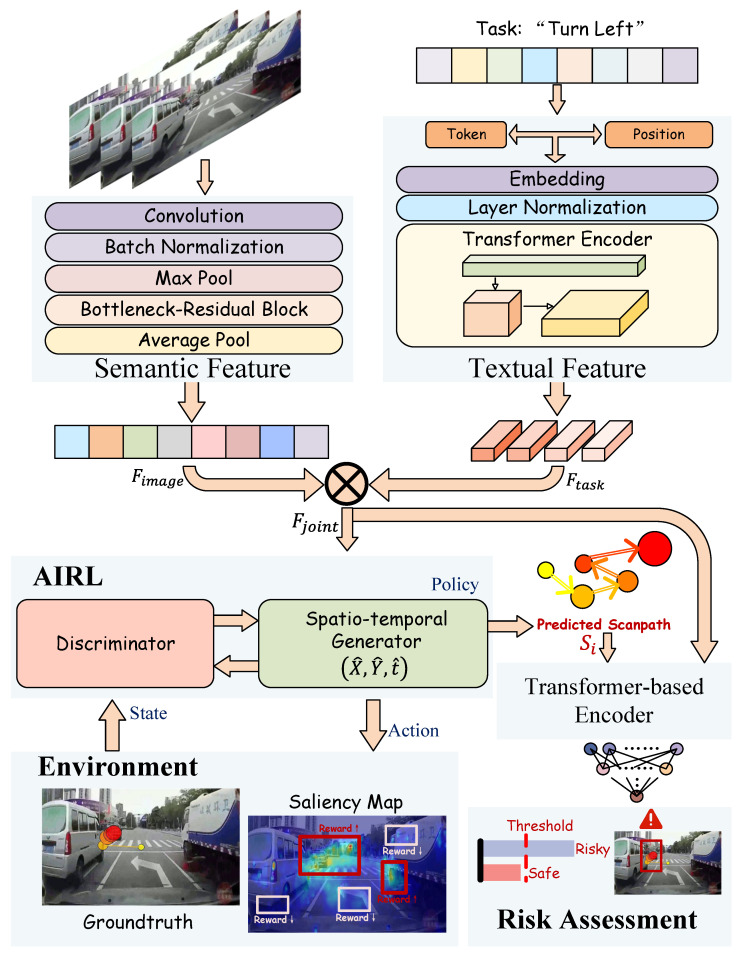
The overview of our cognitive architecture. Semantic features $F_{image}$ from driving scenes and textual features $F_{task}$ of driving tasks yield $F_{joint}$, processed by the AIRL module to derive driver gaze pattern $S_{i}$, and jointly encoded via a Transformer-based encoder for traffic risk perception. Regarding the latent reward, for instance, the sudden appearance of a pedestrian leads to high saliency in the regions corresponding to this safety-critical object. Consequently, the model receives a higher reward when a predicted fixation aligns with these areas.

Our proposed computational framework, shown in Figure 2, is designed to implement the Active Vision hypothesis in driving scenarios. Unlike traditional single-task models, this multi-task learning framework jointly predicts drivers’ scanpaths and assesses traffic risk. The framework simulates three stages of driver cognition:

- **Perception-Goal Integration:** Visual stimuli ($F_{image}$) and top-down task instructions ($F_{task}$) are encoded and fused into a unified cognitive state representation $F_{joint}$, mimicking the brain’s integration of bottom-up cues and top-down goals.
- **AIRL for Scanpath Prediction:** Based on the AIRL paradigm, the model learns a sequential policy to generate scanpaths. Here, the generator acts as the driver’s gaze planner, while the discriminator approximates the driver’s internal safety reward function.
- **Information-Based Risk Perception:** The predicted scanpath $S_{i}$ is not only as a final output but also as an information-gathering process. It is fed into a Transformer-based encoder along with scene features to compute the probability of traffic risk, reflecting how visual attention serves the downstream goal of safe driving.

### 3.2. Scanpath Prediction

The fused representation $F_{joint}$ serves as the driver’s integrated cognitive state, synthesizing bottom-up environmental semantics with top-down task goals. This state is provided to the AIRL framework, where the objective is to generate a sequence of fixations along with their corresponding durations. It reflects how drivers actively sample the driving scenario to maximize information gain under safety constraints.

#### 3.2.1. Generator

The generator *G* serves as the computational model of the driver’s visual search policy π(a,t|S). Instead of passively processing pixels, it acts as an active agent that sequentially decides the next fixation *a* and its duration *t* based on the current cognitive state *S*.

Inspired by [23], the state *S* consists of a saliency map and a fixation-duration mask map.

(1) $$\begin{array}{c} S_{t}=\left\{\begin{array}{cc} L & \;\mathrm{if}\;t=0,\\ M_{t}⊙H+\left(1-M_{t}\right)⊙F_{t-1} & \;\mathrm{otherwise}. \end{array}\right. \end{array}$$

The high-resolution attention map *H* corresponds to the ground-truth attention map, while the low-resolution attention map *L* is obtained by downsampling *H*. $F_{t}$ and $M_{t}$ denote the fovea vision and fixation-duration mask at $t_{\mathrm{th}}$ time step, respectively. The state $S_{t}$ is updated after each selection of a fixation.

The generator *G* predicts a sequence of gaze points ${\left(\left({\hat{X}}_{i},{\hat{Y}}_{i},{\hat{t}}_{i}\right)\right)}_{i=1}^{n}$, where each point corresponds to a state-action pair. *G* aims to maximize the cumulative reward R(S,a) provided by the discriminator *D*. Its loss function $L_{G}$ is defined as follows:

(2) $$\begin{array}{c} L_{G}=-E_{g}\left[R\left(S,a\right)\right], \end{array}$$

(3) R(S,a)=log(D(S,a)),

where $E_{g}$ denotes the expectation based on the sampling from *G*.

#### 3.2.2. Discriminator

The discriminator *D*, implemented as a CNN, distinguishes between real driver scanpaths and those generated by *G*. It estimates the probability D(S,a) that a pair (S,a) originates from the real data distribution. *D* is optimized by minimizing the following cross-entropy loss:

(4) $$\begin{array}{c} L_{D}=-E_{r}\left[\log D(S,a)\right]-E_{g}\left[\log\left(1-D(S,a\right)\right)], \end{array}$$

where $E_{r}$ represents the expectation of sampling from real data.

### 3.3. Traffic Risk Perception

Upon obtaining the predicted driver scanpath $S_{i}$ (*i* stands for the *i*-th driving image), A Transformer-based module combines it with $F_{joint}$ to conduct the final traffic risk assessment. This step models traffic risk assessment not as a static classification, but as a dynamic process where risk perception emerges from the accumulation of visual cues.

#### 3.3.1. Feature Fusion & Encoding

To mimic the brain’s integration of “where” (scanpath) and “why” (risk perception) pathways, we employ a specialized encoding scheme. For each driving frame, $S_{i}$ is normalized by Layer Normalization (LN) to obtain $S_{i}^{\prime }$. A classification token $S_{\mathrm{class}}$ is appended to aggregate global sequence information and yield a fixed-dimensional representation within the Transformer-based encoder.

Crucially, we incorporate positional encoding $E_{\mathrm{pos}}$ to capture the temporal relationships in gaze behavior. This is essential because, in cognitive terms, the *order* of information acquisition (e.g., seeing a car *before* it brakes vs. *after*) fundamentally alters risk interpretation. The input $I_{\mathrm{init}}$ to the Transformer encoder represents the content of the driver’s visual working memory:

(5) $$\begin{array}{c} I_{\mathrm{init}}=\left[S_{\mathrm{class}\;};S_{1}^{\prime };S_{2}^{\prime };\ldots ;S_{N}^{\prime }\right]+E_{\mathrm{pos}}, \end{array}$$

where *N* denotes the number of frames corresponding to each video. Each video frame corresponds to a scanpath.

The scanpath is embedded into a Lyr-layers Transformer encoder, where each layer *l* consists of the multi-head self-attention (MSA) mechanism and Multilayer Perceptron (MLP). The outputs of the MSA module $I_{\mathrm{MSA}}^{l}$ and the MLP module $I_{\mathrm{MLP}}^{l}$ are given below:

(6) $$\begin{array}{c} I_{\mathrm{MSA}}^{l}=\mathrm{MSA}\left(\mathrm{LN}\left(I_{\mathrm{MLP}}^{l-1}\right)\right)+I_{\mathrm{MLP}}^{l-1}, \end{array}$$

(7) $$\begin{array}{c} I_{\mathrm{MLP}}^{l}=\mathrm{MLP}\left(\mathrm{LN}\left(I_{\mathrm{MSA}}^{l}\right)\right)+I_{\mathrm{MSA}}^{l}. \end{array}$$

The final output of Transformer Encoder is defined as:

(8) $$\begin{array}{cc} I_{Lyr}=I_{\mathrm{MLP}}^{Lyr}= & \;\mathrm{MLP}\left(\mathrm{LN}\left(I_{\mathrm{MSA}}^{Lyr}\right)\right)\\ \; & +\mathrm{MSA}\left(\mathrm{LN}\left(I_{\mathrm{MLP}}^{Lyr-1}\right)\right)+I_{\mathrm{MLP}}^{Lyr-1}. \end{array}$$

#### 3.3.2. Risk Assessment

The final stage is information-based risk perception. The token vector for classification $I_{Lyr}^{0}$ in $I_{Lyr}$ serves as a holistic representation of the driving scenario and is fed into an MLP classifier with a softmax activation function to estimate the probability of traffic risk.

The downstream task of traffic risk perception is optimized using cross-entropy loss. The corresponding loss function is defined as follows:

(9) $$\begin{array}{c} L_{accident}=-\sum_{i=1}^{2}y_{i}\log P\left(y=i|I\right),\;i\in \left\{0,1\right\}. \end{array}$$

A scenario is labeled as safe when i=0, and as risky when i=1.

In summary, the overall loss function of the proposed method is:

(10) $$\begin{array}{c} L_{loss}=L_{G}+L_{D}+L_{accident}. \end{array}$$

## 4. Experiments

### 4.1. Dataset

To the best of our knowledge, no public dataset currently integrates spatio-temporal driver eye movements with explicit traffic risk annotations. To bridge this gap, we introduce BDDA, a novel benchmark comprising 1474 class-balanced frames and over 13,000 gaze points. The dataset aggregates risky scenarios from DADA-Diverse [36] and safe scenarios from BDDX-Diverse [37]. To ensure cognitive fidelity, volunteers were instructed to imagine themselves performing real driving under specific task constraints (e.g., accelerate, turn right and go straight). Data collection employed rigorous methodologies to ensure diversity, involving 5 annotators per frame for risky scenarios and over 6 annotators per frame for safe scenarios. Crucially, this dataset captures gaze dynamics across varying risk levels, providing a rigorous basis for analyzing the coupling between visual search and risk perception.

### 4.2. Implementation Details

We resize input images to 512×320 and set the patch size to 16×16. The length of the scanpath is 5. The number of layers for both *G*’s Transformer encoder and decoder is set to 6. We optimize the model using AdamW with disjoint parameter groups (inspired by [21]). Specifically, the learning rates are set to $5\times 10^{-7}$ $1\times 10^{-6}$, and $5\times 10^{-5}$ for the encoder head, duration head, and decoder heads, respectively. We use a batch size of 32 and train the model for 400 epochs. The entire model is trained on an NVIDIA RTX3090Ti GPU. We employ the widely recognized ScanMatch [38] and MultiMatch [39] as evaluation metrics to measure the similarity between the model’s predicted scanpaths and the drivers’ ground-truth scanpaths. For the traffic risk perception task, we employ the commonly used Accuracy (ACC), the Area Under the Curve (AUC), and the F1-score to compare the performance of different models.

### 4.3. Quantitative Analysis

We first evaluate the biological fidelity of the generated scanpaths using the BDDA benchmark. As shown in Table 1, our proposed AIRL-based framework achieves superior performance across nearly all metrics compared to state-of-the-art baselines. In particular, on the ScanMatch metric, which evaluates the spatio-temporal alignment between the predicted and ground-truth gaze sequences, our method scores 0.414 (w/o Dur) and 0.375 (w/ Dur). This represents a substantial improvement of nearly 10% compared to the strongest baseline (Gazeformer). From a cognitive perspective, this indicates that our model does not merely memorize static saliency maps but successfully captures the sequential decision-making process of human vision.

**Figure 3:**
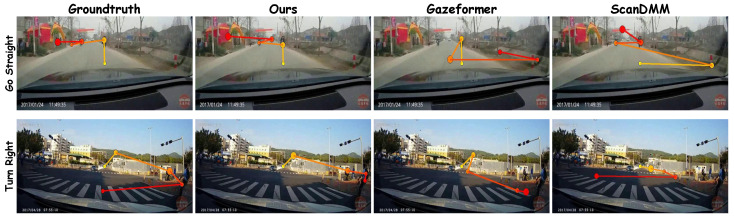
Visualization comparison of scanpaths predicted by different models. The scanpath is represented by circles progressing from yellow (initial fixation) to red (final fixation), where larger radii indicate longer fixation durations.

Results on MultiMatch metrics further reveal the source of this advantage. Our model achieves the highest scores in Vector (0.914) and Direction (0.749). As these metrics are particularly sensitive to saccadic dynamics, the results suggest that our model effectively reproduces human visual orienting mechanisms—capturing not only “where” to gaze, but also “how” attention shifts in response to dynamic driving scenarios. In contrast, baseline models like ScanDMM rely on probabilistic state transitions to model gaze dynamics. While effective for free-viewing, they lack an explicit internal reward mechanism to encode specific driving goals. Consequently, they often fail to capture the precise, task-driven directional shifts required for safety, leading to more dispersed or uncertain gaze patterns, as evidenced by the drifting scanpaths in Figure 3.

### 4.4. Qualitative Behavioral Analysis

To understand the cognitive plausibility of our model in real-world settings, we visualize the predicted scanpaths in safety-critical scenarios (Figure 3). A striking example is observed in the “Turn Right” task. The ground-truth scanpath (Human) exhibits a clear goal-directed attention strategy: the driver ignores the salient buildings in the center and rapidly foveates on the cyclist and pedestrians crossing from the right.

Baseline models (e.g., Gazeformer, ScanDMM) often succumb to bottom-up saliency, gazing at the high-contrast central road or background clutter, failing to account for the task constraint. In contrast, our model successfully replicates the human top-down modulation. Driven by the learned latent reward function, it directs attention towards the task-relevant elements (the cyclist) on the periphery. This qualitative alignment confirms that our AIRL framework has internalized the concept of *risk-aware visual search*. It prioritizes semantically meaningful threats such as cyclists or crossing pedestrians over visually salient distractions. By explicitly gathering visual evidence of these specific hazards, the generated scanpaths effectively serve the downstream goal of risk assessment. This functional equivalence strongly validates the cognitive plausibility of the Active Vision hypothesis.

### 4.5. The Coupling of Attention and Risk Perception

We investigate whether this improved scanpath modeling leads to better risk understanding. As detailed in Table 2, our unified framework achieves superior performance on the proposed benchmark in traffic risk assessment, with an ACC of 0.883 and an AUC of 0.912. Notably, our method outperforms models that do not explicitly model eye movements (e.g., DRIVE, DSTA) by significant margins (∼17% and ∼9%, respectively). This empirical evidence strongly supports the *Active Vision hypothesis*: accurate risk perception is contingent upon the active sampling of relevant visual information. Furthermore, compared to THAT-Net, which uses a more complex network but lacks our explicit scanpath-risk coupling, we still achieve a ∼2% improvement in accuracy. This reinforces the idea that a *selective attentional bottleneck* is crucial: by enforcing the model to look at “human-like” regions, we effectively filter out noise and provide the risk assessment module with high-fidelity and evidence-based visual representations.

### 4.6. Ablation Study

To assess the effectiveness of the proposed modules, we conduct the ablation study to examine the contribution of each feature to the model’s performance.

We first investigate the impact of components $F_{image}$, $F_{task}$, and $S_{i}$ on the performance of the traffic risk perception method (Table 3). Removing semantic features $F_{image}$ or textual task features $F_{task}$ results in moderate performance drops (ACC decreases by ∼2.2% and ∼2.4%, respectively). This suggests that while bottom-up visual cues and top-down task instructions provide the necessary context, they are insufficient on their own to fully explain driver behavior.

Crucially, $S_{i}$ proves to be the most influential feature, the absence of scanpath information leads to a sharp decline in ACC (0.883 → 0.835, a ∼5% drop) and F1-score (0.879 → 0.828). It indicates that gaze behavior acts as a critical *information bottleneck*. Without the explicit guidance of “where” the driver is looking, the risk perception module is forced to process the entire visual scene uniformly, likely becoming overwhelmed by irrelevant visual noise. The integration of spatio-temporal eye movement mechanisms allows the model to selectively attend to safety-critical regions, thereby constructing a more accurate and efficient representation of risk.

Having established that *looking* is essential for risk assessment, we further ask: does the *quality* of the visual search policy matter? To answer this, we replace our AIRL-based generator with other state-of-the-art scanpath prediction models and evaluate how well their generated scanpaths support downstream risk perception.

Table 4 reveals a clear correlation between the behavioral fidelity of the scanpath and the accuracy of risk perception. Stochastic or purely saliency-based models, such as PathGAN (ACC 0.677), yield significantly lower performance. This is likely because these models are designed primarily for free-viewing tasks and fail to capture the highly goal-directed nature of driving. Their generated gaze patterns tend to be dispersed, failing to provide the “evidence” needed for risk evaluation.

Goal-oriented models like Gazeformer achieve respectable performance (ACC 0.854), confirming that incorporating task constraints is beneficial. However, our proposed AIRL framework still outperforms all baselines (ACC 0.883), achieving an improvement of approximately 2% over the strongest competitor. This superiority can be attributed to the Inverse Reinforcement Learning paradigm. Our approach recovers the driver’s latent reward function, i.e., the internal valuation of safety. This allows our model to generate scanpaths that are not just geometrically similar to human data but functionally equivalent in their ability to sample risk-relevant information.

### 4.7. Practical Efficiency Analysis

To evaluate the feasibility of deploying our model in real intelligent driving systems, we analyze its computational cost and theoretical real-time efficiency. Specifically, the proposed model maintains a moderate computational complexity of 23.63 GFLOPs. This restricted computational footprint significantly reduces the hardware burden, ensuring that the model can operate with high efficiency. Given that emergency braking scenarios place stringent demands on processing speeds, the lightweight nature of our architecture provides a solid foundation for real-time readiness, making it highly suitable for seamless integration into Advanced Driver Assistance Systems (ADAS) or autonomous vehicle pre-warning modules.

## 5. Discussion

Our results provide convincing empirical support for the Active Vision hypothesis within the domain of driving. By coupling scanpath prediction and risk perception into a unified computational framework, we demonstrate that these two processes are not merely statistically correlated but mechanistically coupled within the driver’s cognitive architecture. Visual attention, in this view, is not a passive reaction to environmental stimuli (bottom-up saliency) but an active, goal-directed information-foraging process essential for the downstream task of risk assessment. This integration suggests that the human visual system strategically samples the environment to reduce uncertainty and gather evidence that is most relevant to safety-critical decision-making.

### 5.1. Cognitive Plausibility of AIRL

Our framework distinguishes itself from a single Generative Adversarial Network (GAN) or Inverse Reinforcement Learning (IRL) by architecting an organic integration of the two. Why is such a hybrid architecture necessary? We consider that in a standard GAN, the discriminator acts solely as a classifier to guide distribution matching. In contrast, our generator is not trained to directly match the data distribution, but to learn a scanpath prediction policy π(a,t|S) that interacts with an evolving environmental state. This policy is optimized via reward maximization, where the learned reward is defined implicitly through the adversarial training process. This mechanism is fundamentally consistent with IRL formulations, recovering the latent reward function that governs human-like visual foraging.

The biological plausibility of our approach lies in shifting from passive saliency to active search. Our AIRL-based generator acts as an agent that sequentially plans fixations to maximize a latent reward. This mirrors the human cognitive process of strategically allocating attention to identify potential hazards. By prioritizing the scanpath generation through AIRL, we ensure that the “where” of looking reflects human-like driving goals before the information is processed for risk assessment.

### 5.2. The “Look-to-Percept” Bottleneck

Our findings elucidate a critical attentional bottleneck that underscores the mechanistic coupling between visual sampling and safety assessment. Under the Active Vision framework, the scanpath generated by our AIRL module functions as a high-fidelity information filter, prioritizing task-relevant stimuli over raw environmental data. This selective process aligns with the principle that human cognition avoids the prohibitive cost of processing high-dimensional visual scenes in their entirety by focusing on sparse but highly informative regions. Without the predicted scanpath, the model’s performance of risk perception drops sharply. This suggests that the “why” of risk perception (the downstream task) is fundamentally dependent on the quality of the “where” (the AIRL output).

By training the model to mimic human gaze through AIRL, we establish a meaningful order for information acquisition. This allows the risk perception module to focus on a sequence of key events, such as a pedestrian stepping out or a car’s brake lights, rather than processing the entire scene at once. This process shows that a scanpath is not just a series of eye movements, but a structured timeline of evidence. A scanpath is not merely a byproduct of vision, but a necessary cognitive support that organizes visual evidence for fast and accurate traffic risk evaluation.

### 5.3. Limitations and Future Work

While our integrated framework demonstrates superior performance on the BDDA benchmark by coupling scanpath prediction with risk perception, the current design has a few weaknesses that require more research. We discuss our limitations in three aspects: how the eyes work, how drivers differ, and how to close the loop between seeing and acting.

**How the eyes work:** While our AIRL-based generator effectively models the deployment of foveal fixations and utilizes a low-resolution map *L* (Equation (1)) as a preliminary multi-scale proxy for peripheral blurriness, it does not yet fully account for the critical active trigger role of peripheral vision [45]. In human cognition, peripheral vision acts as a high-sensitivity monitor for sudden-onset stimuli, which subsequently triggers the saccades predicted by our model. Future research should explore a dual-stream architecture incorporating explicit eccentricity-dependent visual processing to model the interplay between ambient and focal vision, allowing the model to simulate how a driver’s latent reward function is dynamically modulated by peripheral “bottom-up” cues in safety-critical scenarios. Additionally, transitioning from static frame processing to continuous video streams in the future will require incorporating velocity-matching control loops and different sensorimotor time scales to accurately model dynamic tracking behaviors like smooth pursuit, thereby achieving higher fidelity to real-world oculomotor behaviors.

**How drivers differ:** Our study is bounded to identifying generic scanpath patterns to establish a universal baseline for general driving safety, which provides essential insights into fundamental human-like risk sampling despite neglecting individual differences [7,22,46]. Real-world driving behavior is deeply influenced by personal risk tolerance and experience. For example, a novice driver may focus more on immediate lane-keeping, while an experienced driver maintains a broader search for potential hazards. Our current model does not distinguish between these different search priorities. Incorporating driver-specific priors or personalized reward functions within the AIRL paradigm could further refine the model, enhance its predictive accuracy across diverse populations and develop customized driver assistance systems.

**How environments vary:** While the BDDA dataset aggregates over 13,000 spatial-temporal gaze points, collecting real eye-tracking data during actual traffic accidents remains unfeasible due to strict safety and ethical constraints. Consequently, our dataset relies on an imagined driving setup under controlled conditions, which nevertheless successfully captures characteristic defensive eye movements such as rapid saccades toward potential hazards. Furthermore, although the BDDA dataset includes diverse scenarios such as night driving, the sample size for these specific conditions is currently smaller than that for day driving. Future iterations will expand data collection to include a more balanced variety of environmental conditions and driving styles to further test the robustness of the model.

**How to close the loop:** The current framework operates as an open-loop system. It predicts perception but does not yet execute motor control. According to the broader Active Vision hypothesis, perception and action are not separate stages but a continuous and reciprocal cycle. In a real car, eye movements are planned to facilitate specific maneuvers like braking or steering, and the results of those actions then guide where the driver looks next. A vital direction for future research involves closing this perception-action loop by integrating our cognitive model with vehicle control modules. Modeling the full cycle from active information foraging to the execution of safety maneuvers is essential for developing autonomous systems that truly align with human cognitive expectations and behavioral patterns.

## 6. Conclusions

In this paper, we introduced a unified framework grounded in Adversarial Inverse Reinforcement Learning to model scanpath prediction and driving risk perception simultaneously. Our approach leverages an adversarial generator to learn a human-like scanpath policy, synergistically integrated into a Transformer for risk assessment. Experiments conducted on our newly proposed BDDA benchmark demonstrate that our approach achieves superior performance on both tasks. Furthermore, our ablation studies confirm the critical role of explicit scanpath modeling in improving risk perception accuracy, confirming the Active Vision hypothesis that perception is mechanistically coupled with attentional allocation. Future work will focus on incorporating peripheral vision mechanisms, modeling individual differences in driver experience, and closing the perception-action loop by integrating this cognitive model with vehicle control modules, to further enhance the model’s contextual understanding in complex driving environments.

## Figures and Tables

**Table 1:** Comparative results of scanpath prediction on the BDDA dataset. **Bold** shows the best performance.

Method	ScanMatch		MultiMatch				
	w/o Dur↑	w/ Dur↑	Vector↑	Direction↑	Length↑	Position↑	Duration↑
PathGAN [40]	0.053	0.046	0.872	0.650	0.867	0.401	0.627
IOR-ROI [17]	0.229	0.227	0.821	0.735	0.802	0.648	0.615
Chen et al. [18]	0.316	0.282	0.889	0.731	0.824	0.709	0.631
Gazeformer [21]	0.374	0.317	0.891	0.744	0.832	0.794	0.621
ScanDMM [41]	0.301	-	0.810	0.613	0.709	0.760	-
SEA [24]	0.318	-	0.893	0.655	0.855	**0.834**	-
**Ours**	**0.414**	**0.375**	**0.914**	**0.749**	**0.889**	0.762	**0.655**

**Table 2:** Comparative results of traffic risk perception incorporating spatio-temporal modeling of eye movement mechanisms (scanpaths). **Bold** shows the best performance.

Method	ACC↑	AUC↑	F1↑
DRIVE [42]	0.716	0.745	0.705
DSTA [43]	0.793	0.816	0.788
THAT-Net [44]	0.869	0.890	0.860
DAA-GNN [33]	0.835	0.847	0.838
**Ours**	**0.883**	**0.912**	**0.879**

**Table 3:** Effect of different features on performance for traffic risk perception. **Bold** shows the best performance.

Method	ACC↑	AUC↑	F1↑
w/o semantic feature	0.861	0.881	0.852
w/o textual feature	0.859	0.878	0.850
w/o scanpath information	0.835	0.869	0.828
**full features**	**0.883**	**0.912**	**0.879**

**Table 4:** Effect of scanpath modeling approaches on performance for traffic risk perception. **Bold** shows the best performance.

Method	ACC↑	AUC↑	F1↑
PathGAN [40]	0.677	0.693	0.670
IOR-ROI [17]	0.809	0.814	0.794
Chen et al. [18]	0.812	0.839	0.804
Gazeformer [21]	0.854	0.870	0.849
ScanDMM [41]	0.754	0.772	0.750
SEA [24]	0.768	0.776	0.763
**Ours**	**0.883**	**0.912**	**0.879**

## Data Availability

The dataset used in this study aggregates risky data from DADA-Diverse [36] and safe data from BDDX-Diverse [37].
